# Non-steroidal anti-inflammatory drugs and pancreatic cancer risk: a nested case–control study

**DOI:** 10.1038/sj.bjc.6605636

**Published:** 2010-04-06

**Authors:** M C Bradley, C M Hughes, M M Cantwell, G Napolitano, L J Murray

**Affiliations:** 1 Cancer Epidemiology and Prevention Research Group, Centre for Public Health, Queen's University, Mulhouse Building, Royal Victoria Hospital, Belfast BT12 6BJ, Northern Ireland, UK; 2 Clinical and Practice Research Group, School of Pharmacy, Queen's University Belfast, 97 Lisburn Road, Belfast BT9 7BL, Northern Ireland, UK; 3 Northern Ireland Cancer Registry, Mulhouse Building, Royal Victoria Hospital, Belfast BT12 6BJ, Northern Ireland, UK

**Keywords:** NSAIDs, aspirin, pancreatic cancer, GPRD

## Abstract

**Background:**

Non-steroidal anti-inflammatory drug (NSAID) use has been linked with pancreatic cancer risk; however, findings from epidemiological studies are inconsistent.

**Methods:**

A nested case–control study was conducted within the UK General Practice Research Database. Cases (*n*=1141) had a diagnosis of primary cancer of the exocrine pancreas between January 1995 and June 2006. Controls (*n*=7954) were matched with each case on general practice site, sex and year of birth. Conditional logistic regression analyses were used to generate odds ratios (OR) and 95% confidence intervals (CI) associated with NSAID use compared with non-use.

**Results:**

Any use of NSAID in the 5 years before the index date or since entry into the database (excluding the year before diagnosis) was not associated with risk of pancreatic cancer; OR 0.96 (95% CI, 0.84–1.10) and 1.03 (95% CI 0.89–1.19), respectively. Exposure to NSAIDs for > 773 days, in the 5 years pre-diagnosis, was associated with a reduced risk of pancreatic cancer OR 0.78 (95%CI 0.62–0.97). There was evidence of reduced pancreatic cancer risk with long-term use (5 years or more) of lower doses of NSAIDs OR 0.70 (95% CI 0.49–0.99).

**Conclusion:**

Long-term exposure to NSAIDs may be associated with a reduction in risk of pancreatic cancer.

Pancreatic cancer is rapidly fatal and is the fifth leading cause of cancer mortality in the western world. (Jemal *et al*, 2007) It is therefore important to identify aetiological and protective factors for this disease to improve primary prevention. Non-steroidal anti-inflammatory drugs (NSAIDs), especially aspirin, have been reported to reduce the risk of several cancers and some pre-malignant lesions, with risk reductions ranging from 20% to 50% for certain cancer sites (Garcia-Rodriguez and Huerta-Alvarez, 2001; Khuder and Mutgi, 2001; Gonzalez-Perez *et al*, 2003; Abnet *et al*, 2009; Cole *et al*, 2009).

However, despite biologically plausible mechanisms whereby NSAIDs may prevent pancreatic cancer, the findings of epidemiologic studies are inconsistent (Schreinmachers and Everson, 1994; Coogan *et al*, 2000; Langman *et al*, 2000; Anderson *et al*, 2002a; Menezes *et al*, 2002; Friis *et al*, 2003; Sorensen *et al*, 2003; Jacobs *et al*, 2004; Ratnasinghe *et al*, 2004; Schernhammer *et al*, 2004; Cook *et al*, 2005). In a recent meta-analysis of these studies, the combined relative risk of pancreatic cancer in regular users of NSAIDs compared with non-regular or never users was 1.01 (95% confidence intervals (CI) 0.91–1.11) (Larsson *et al*, 2006). The Women's Health Study randomised controlled trial reported similar null findings (Cook *et al*, 2005). The inconsistency between laboratory and epidemiological evidence prompted us to examine the association between NSAIDs and pancreatic cancer risk in the UK General Practice Research Database (GPRD), particularly because most studies have been undertaken within the US, (Capurso *et al*, 2007) where NSAIDs are used at higher doses and for longer durations than in the UK (Arellano *et al*, 2006).

## MATERIALS AND METHODS

We undertook a nested case–control study within the UK GPRD, which was established in 1987, and is the world's largest computerised database of anonymised longitudinal patient records from primary care. It collects data from around 500 general practices in the UK, covers about 5.5% of the population and is broadly representative of the UK population (GPRD, 2009). Participating practices follow protocols for the recording and transferring of computerised data to the research database (Hollowell, 1997). The data obtained are assessed in terms of completeness, continuity and plausibility and are from general practices that meet predefined standards, registered as ‘up to standard’ (UTS) practices. GPRD data includes demographic information, clinical diagnoses, referral information, specialty consultation notes, results of laboratory tests and hospital discharge information. Read and Oxford Medical Information System (OXMIS) codes are used to classify medical diagnoses. The GPRD also contains details of all prescriptions issued for registered patients (Garcia Rodriguez and Perez Gutthann, 1998). The high quality of GPRD prescription and diagnosis information has been documented (Jick *et al*, 1991; Jick *et al*, 1992; Van Staa and Abenhaim, 1994). Ethical approval for all observational research using GPRD data has been obtained from a Multicentre Research Ethics Committee.

The study included patients from UTS practices with diagnostic codes for primary pancreatic cancer, with a date of diagnosis/index date (first recorded occurrence of a pancreatic cancer code) between January 1995 and June 2006; data were extracted from GPRD in February 2007. Patients aged 85 or older were excluded, as were cases without at least 5 years of UTS data collection before the index date. Controls with no GPRD record of pancreatic cancer were selected using incidence density sampling and matched to cases on year of birth, sex and general practice site. Controls also had at least 5 years of UTS data collection before their index date (date of pancreatic cancer diagnosis in their matched case). Up to seven controls were matched for each case.

Pancreatic cancer is difficult to conclusively diagnose without histological or cytological examination of relevant specimens and these specimens are often not available. Pancreatic cancer may therefore be confused with primary malignancies in adjacent organs or with secondary tumours. Histological confirmation was not available from GPRD as there was no routine linkage to cancer registries, at the time the data were obtained. To minimise misclassification, all cancer codes recorded in the medical records of potential cases were reviewed. Only cases with consistently recorded pancreatic cancer codes were included. All other cases, for example patients with codes for both pancreatic cancer and cholangiocarcinoma, or carcinoma of bile duct, gallbladder and so on, and their matched controls were excluded from the dataset; peri-ampullary tumours were also excluded. Cases and controls with a prior history of cancer (at least a year before the index date) were identified by review of cancer codes and flagged as having a previous cancer but were not excluded from the dataset.

The primary exposure of interest was NSAID use in the 5 years before the index date, excluding the year before the index date. NSAID use in the year before diagnosis was excluded because early cancer symptoms may have led to NSAID use during this period. NSAID use since entry into GPRD until a year before the index date was also examined. Data were extracted on all NSAIDs prescribed for cases and controls. NSAID use was expressed in units of defined daily dose (DDD), a validated measure of drug consumption maintained by the World Health Organisation (WHO). It is the assumed average maintenance dose per day of a drug used for its main indication in adults. (World Health Organisation Working Group, 2008) As the DDD of a drug may be assumed to be functionally equivalent to the DDD of any other drug used for a similar purpose, DDDs may be used to compare or combine the usage of drugs within and across pharmacological groups, for example all NSAIDs.

The number of DDDs for each NSAID prescription issued was calculated by multiplying the dose prescribed by the quantity given and dividing this by the DDD value assigned to that drug. For each time period under study, the total number of DDDs for each NSAID prescribed, for subgroups of NSAIDs (aspirin and derivatives, high-dose aspirin (300 mg or more a day), fenamates, propionic acid derivatives, acetic acid derivatives, COX-2 inhibitors, oxicams or others), and for all NSAIDS combined was calculated for each case and control. Cumulative duration of any NSAID use was calculated as the total number of intended days, during the periods under study, for which study subjects were issued any prescription (at any dose) for NSAIDs. Total dose (DDDs) and total duration (days) of any NSAID use, in the periods under study, were categorised into approximate quartiles, on the basis of total NSAID use within the controls.

Exposure to any NSAID at any time before the index date, excluding the year before the index date, was further categorised according to both dose and duration of use combined. The median number of DDDs, per day, of NSAID exposure in the controls during this period was one DDD per day, therefore low-dose NSAID exposure was classified as the use of <1.0 DDD of NSAIDs per day and high dose as >1.0 DDDs per day. Categories for duration of low and high NSAID use were subsequently created: no use or use for <1 year (0–365.3 days), use for 1–3 years (365.3–1095.8 days), use for 3–5 years (1095.8–1826.3 days) and use for 5 or more years (>1826.3) (Yang *et al*, 2007).

### Statistical analysis

Conditional logistic regression was used to calculate odds ratios (ORs) and 95% percent CIs for the associations between disease status and use of NSAIDs.

Models were constructed for ever/never use of all NSAIDs or subgroups of NSAIDs. Dose and duration of NSAID use was examined using continuous variables (output expressed per 200 DDDs and 200 days of use), approximate quartiles of dose and duration of NSAID use and the combined dose and duration variable. NSAID use in the year before diagnosis was excluded in all analyses and models were constructed relating to the 5-year period before diagnosis/index date and since entry into the GPRD.

All analyses were adjusted for potential confounders including smoking status (unknown, current smoker, non-smoker, ex-smoker), body mass index (BMI, unknown, underweight, normal, overweight, obese according to WHO categories) (World Health Organisation Working Group, 2009), alcohol use (unknown, current drinker, lifelong non-drinker, ex-drinker), history of pancreatitis, history of rheumatoid arthritis, history of diabetes and history of cancer. For smoking, alcohol use and BMI, we used the most recent record excluding those within the year before the index date and for history of diabetes, pancreatitis and cancer we also excluded events within the year before the index date. We also adjusted for use of H2 antagonists (ever/never use) or proton pump inhibitors (PPIs, ever/never use), systemic steroids (ever/never use), hormone replacement therapy (HRT, ever/never use) and disease modifying anti-rheumatic drugs as use of these drugs may confound the association between NSAIDs and pancreatic cancer. Use of HRT and steroids in the year before diagnosis was excluded. As there was a strong positive association between use of H2 antagonists and PPIs in the 2 years before diagnosis (presumably because of cancer-associated symptoms) use of these drugs within this period was excluded. Data on the confounders were not available for all subjects and for those subjects for whom all data was available, a restriction analyses was carried out. STATA Version 9 (Timberlake Consultants Ltd, London, UK) was used for all analyses.

## RESULTS

Within the GPRD, 1361 pancreatic cancer cases met the initial inclusion criteria and these were matched to 9487 controls. After review of cancer codes, 220 cases (16%) and their controls (1542, 9.4%) were excluded, leaving 1141 pancreatic cancer cases and 7954 matched controls. Each case had at least one matched control and >90% of cases had seven matched controls. The mean duration of UTS follow-up was 10.6 (s.d. 3.4) years, for both cases and controls. The mean age at database enrolment was also the same for cases and controls at 57.3 (s.d. 9.8) years; over 50% (53.7%) of all subjects were male (Table 1).

Any use of an NSAID in the 5 years before the index date or since entry into the GPRD (excluding the year before diagnosis) was not associated with risk of pancreatic cancer; (OR) 95% CI, 0.96 (0.84–1.10) and 1.03(0.89–1.19), respectively (Data not shown). Any use of aspirin and its derivatives or high-dose aspirin in the 5 years before the index date was also not associated with pancreatic cancer risk: ORs (95% CI), 0.95 (0.81–1.13) and 0.91 (0.61–1.35), respectively, and similar results were seen when use since entry into the GPRD was examined (Table 2). Among subgroups of NSAIDs, risk of pancreatic cancer was lowest among users of COX-2 inhibitors and oxicams but the reductions in risk did not reach statistical significance; adjusted ORs (95% CI), 0.80 (0.56–1.11) and 0.67 (0.43–1.03), respectively for use in the 5 years before diagnosis. Use of other subgroups of NSAIDs (acetic acid derivatives, fenamates, propionic acid derivatives or other NSAIDs) was not associated with pancreatic cancer risk (Table 2). There was also no overall association between pancreatic cancer risk and the total dose of NSAIDs used in the 5 years before the index date or since entry into the GPRD (excluding the year before diagnosis). The adjusted ORs (95% CI) for an increase in 200 DDDs of NSAIDs in these two periods were 0.99 (0.94–1.03) and 0.99 (0.97–1.01), respectively (Data not shown). When the dose of NSAIDs was categorised according to use in controls (approximate quartiles) no association was seen between dose category and pancreatic cancer risk (Table 3).

There was an inverse association between pancreatic cancer risk and duration of NSAID use in the 5 years before the index date. NSAID use for 773 days or more (approximately 2 years) in this period (excluding the year before the index date) was associated with a decrease in risk; adjusted ORs (95% CI), 0.78 (0.62–0.97) (Table 4). No association was seen in the period since entry into the GPRD (Table 4). Results of the analysis that combined dose and duration of NSAID use since entry into GPRD (excluding the year before the index date) are shown in Table 5. There was also some evidence in this analysis of reduced pancreatic cancer risk with long-term use (use for 1826.3 days or more) of lower doses of NSAIDs (<1 DDD a day); adjusted ORs (95% CI), 0.70 (0.49–0.99).

## DISCUSSION

In this large population-based prospective study no significant reductions in risk of pancreatic cancer were seen for ever compared with never use of an NSAID. There was also no overall association between risk and the total dose prescribed and no risk reduction was seen among subjects who used the highest doses of NSAIDs. A 20% reduction in risk was seen among subjects who had been prescribed an NSAID for approximately 2 years or longer in the 5 years before diagnosis and a 30% reduction in subjects who had used lower than average doses of NSAIDs for 5 years (1826.3 days) or more since entry into the GPRD. However, no consistent pattern of reduced risk was seen with increasing dose, duration or combined dose and duration of NSAID use. Modest reductions in long-term users of higher doses of NSAIDs were seen but were not statistically significant. Although chance and residual confounding may explain the latter findings, these data suggest that long-term use (>2 years) of NSAIDs (at regular anti-inflammatory doses) may protect against pancreatic cancer and that duration of use may be more important than dose.

Two recent meta-analyses found no association between aspirin/NSAID use and pancreatic cancer. (Larsson *et al*, 2006; Capurso *et al*, 2007). Capurso *et al* (2007) reported pooled ORs (95% CI) for pancreatic cancer risk of 0.99 (0.83–1.19), 1.11 (0.84–1.47) and 1.09 (0.67–1.75) in low, intermediate and high aspirin/NSAID exposure groups, respectively (based on a combination of dose and duration). Duration of use was not examined separately from dose in this meta-analysis. A previous study using the GPRD, which included fewer pancreatic cancer cases (513) than our study, reported an OR of 1.49 (1.02–2.18) for patients receiving 7 or more prescriptions for NSAIDs in the 13–36 months before the diagnosis date of the case (Langman *et al*, 2000). This study may have comprised cases also included in our analysis, but we believe our study was more rigorous as it examined both the dose and duration of NSAID exposure rather than the number of NSAID prescriptions issued; similarly, the UTS follow-up time (a minimum of 5 years) and the median follow-up time (10.6 years) in our study were longer than in the earlier study (at least 3 years). Moreover, the risk estimates reported in that study were adjusted only for age and smoking status, whereas we applied a comprehensive range of adjustments.

Ever use of aspirin was not associated with pancreatic cancer risk in our study, which is not unexpected as the majority of aspirin users (83%) used low-dose aspirin only (<300 mg) and data from cohort studies (Friis *et al*, 2003) and large trials have not shown any decrease in pancreatic cancer risk in users of low-dose aspirin (Cook *et al*, 2005). Use of greater than five aspirin tablets per week (high dose, 325 mg) for 10 years or more in the Nurses’ Health Study was associated with an increased the risk of pancreatic cancer compared with those who used aspirin for 1–5 years (Schernhammer *et al*, 2004). Data from the two large randomised trials, with reliable post trial follow-up for more than 20 years, also suggested that high-dose aspirin may increase the risk of pancreatic cancer slightly (Flossmann *et al*, 2007). We did not see any increase in risk in subjects using high-dose aspirin but we were unable to assess risk in long-term users of high-dose aspirin as only 17% used this dose at any time.

Ever versus never use of COX-2 inhibitors and oxicams was associated with modest reductions in pancreatic cancer risk (20% and 30%, respectively) but statistical significance was not achieved and too few subjects took these preparations to allow a more detailed analysis of use of these drugs. Trials of COX-2 inhibitors in patients with colorectal adenomas or previous colorectal cancer have shown that these drugs can successfully prevent colorectal carcinogenesis (Arber *et al*, 2006; Baron *et al*, 2006; Bertagnolli *et al*, 2006) but adverse effects on cardiovascular risk mean that they are also unlikely to be of benefit for the prevention of cancer, especially if a high-risk pre-malignant state is not clearly recognised (Lynch, 2006). Meloxicam has been described as a preferential COX-2 inhibitor and as with piroxicam has shown inhibitory actions on human cancers (Ding *et al*, 2003; Naruse *et al*, 2006). This is the first study to report on the use of oxicams and pancreatic cancer prevention and further analysis is warranted.

This analysis has several key strengths, being the largest and most detailed of the subject to date, allowing us to stratify the analyses by dose, duration and subgroup of NSAID. The use of prospectively collected prescription data avoids errors of recall and potential recall bias associated with questionnaire-based measures of NSAID use. Moreover, all subjects in our study had at least 5 years of data available before diagnosis and data were available before diagnosis for a mean of over 10 years. We also adjusted for all major confounders and, although data were not available for all subjects, the results of restriction analyses including only those patients who had data on these confounders were not different from the main analyses. However, we cannot rule out residual confounding. We do not believe that confounding by indication is a major problem within the study as indications for which NSAIDs are used are very varied and it is unlikely that each indication would have a common association with pancreatic cancer risk. We attempted to address such confounding by adjusting for a history of rheumatoid arthritis, a common indication of chronic NSAID use; it made no difference to observed ORs.

This study has some limitations. Data on prescriptions issued may not reflect actual use of NSAIDs but there is no reason to believe that non-compliance with prescription medication would differ systematically between cases and controls. No information was available on over-the-counter (OTC) NSAID use (including that from non-pharmacy outlets) but there is little evidence of large-scale OTC purchasing of NSAIDs in the UK, especially in the middle aged and elderly. (Meier *et al*, 2002) Pancreatic cancer diagnoses were not validated and some misclassification of diagnoses may have occurred within the GPRD system but we excluded very elderly subjects, in which diagnostic accuracy may be a particular problem; also, all cancer codes were reviewed and cases with inconsistent coding were excluded. Furthermore, cancer diagnoses in GPRD appear to be a reliable record of incident cancer diagnoses and have been shown to concord with original medical records in 95% of cases. (Jick *et al*, 1997)

In summary, this study suggests that long-term use (>2 years) of NSAIDs may protect against pancreatic cancer but replication of these findings is required.

## Figures and Tables

**Table 1:** Characteristics of cases and controls

	**Cases (*n*=1141)**	**Controls (*n*=7954)**	***P* value**
Age at database enrolment (years, s.d.)	57.3 (9.8)	57.3 (9.8)	1.0
Male sex (%)	53.7	53.7	1.0
Mean duration of follow-up before index date (years, s.d.))	10.6 (3.4)	10.6 (3.4)	1.0
*Smoking status*			<0.005
Current smoker	302 (26.5%)	1372 (17.3%)	
Non-smoker	463 (40.6%)	3961 (49.8%)	
Ex-smoker	235 (20.6%)	1499 (18.9%)	
Missing data	141 (12.4%)	1122 (14.1%)	
*Body mass index (kg m* ^*−2*^ *)*			0.1
<18.5	16 (1.4%)	102 (1.3%)	
⩾18.5 & <25	353 (31.0%)	2323 (29.2%)	
⩾25 & <30	343 (30.1%)	2525 (31.8%)	
⩾30	187 (16.4%)	1108 (13.9%)	
Missing	242 (21.2%)	1896 (23.8%)	
*Current drinking status*			0.4
Current drinker	731 (64.1%)	4992 (62.8%)	
Non-drinker	165 (14.5%)	1107 (13.9%)	
Ex-drinker	7 (0.6%)	77 (1.0%)	
Missing data	238 (20.9%)	1778 (22.4%)	
*Hormone replacement therapy use*			0.3
Never	1095 (95.9%)	7574 (95.2%)	
Ever	46 (4.1%)	380 (4.8%)	
*Steroid use*			0.2
Never	990 (86.8%)	6999 (88.0%)	
Ever	151 (13.2%)	955 (12.0%)	
*Pancreatitis*			<0.005
Never	1085 (95.1%)	7901 (99.3%)	
Ever	56 (4.9%)	53 (0.7%)	
*Proton pump inhibitor use*			0.3
Never	964 (84.5%)	6817 (85.7%)	
Ever	177 (15.5%)	1137 (14.3%)	
*H*_*2*_ *antagonist use*			<0.005
Never	877 (76.9%)	6423(80.8%)	
Ever	264 (23.1%)	1531 (19.2%)	
*Diabetes*			<0.005
Never	1006 (88.2%)	7442 (93.5%)	
Ever	135 (11.8%)	512 (6.4%)	
*Prior cancer*			0.4
Never	1049 (91.9%)	7374 (92.7%)	
Ever	92 (8.1%)	580 (7.3%)	
*Rheumatoid arthritis*			
RA codes			0.2
Never	1131	8993	
Ever	10	102	
Disease modifying anti-rheumatic drugs prescription			0.9
Never	1126	8993	
Ever	15	102	

**Table 2:** Ever use of different groups of non-steroidal anti-inflammatory drugs (NSAID) in the two time periods

	**Five years before one index date**				**Until 1 year before the index date**			
**Odds ratios for ever/never use**	**Cases (*n*=1141)**	**Controls (*n*=7954)**	**OR (95%CI)**	**Adjusted OR (95%CI)** a	**Cases (*n*=1141)**	**Controls (*n*=7954)**	**OR (95%CI)**	**Adjusted OR (95%CI)** a
Acetic Acid derivatives	249 (21.8%)	1747 (22.0%)	0.99 (0.85–1.16)	0.96 (0.82–1.13)	409 (35.9%)	2762 (34.7%)	1.06 (0.92–1.21)	0.99 (0.86–1.14)
Aspirin and derivatives	236 (20.68%)	1601 (20.1%)	1.03 (0.88–1.22)	0.95 (0.81–1.13)	267 (23.4%)	1813 (22.8%)	1.04 (0.89–1.21)	0.95 (0.81–1.12)
Coxibs	45 (3.94%)	353 (4.4%)	0.87 (0.63–1.22)	0.80 (0.56–1.11)	47 (4.1%)	356 (4.5%)	0.91 (0.66–1.26)	0.82 (0.58–1.16)
Fenamates	14 (1.2%)	93 (1.2%)	1.06 (0.59–1.89)	1.02 (0.56–1.86)	43 (3.8%)	267 (3.4%)	1.14 (0.81–1.60)	1.09 (0.77–1.55)
Other NSAIDs	19 (1.7%)	86 (1.1%)	1.57 (0.95–2.60)	1.43 (0.85–2.43)	44 (3.9%)	219 (2.75%)	1.44 (1.03–2.01)	1.41 (1.0–1.99)
Oxicams	24 (2.1%)	234 (2.9%)	0.71 (0.46–1.08)	0.67 (0.43–1.03)	62 (5.4%)	480 (6.0%)	0.89 (0.68–1.18)	0.83 (0.62–1.10)
Propionic acid derivatives	307 (26.9%)	2023 (25.4%)	1.08 (0.94–1.25)	1.06 (0.92–1.23)	574 (50.3%)	3715 (46.7%)	1.17 (1.03–1.32)	1.12 (0.98–1.28)
High-dose aspirin	30 (2.6%)	208 (2.6%)	1.0 (0.67–0.48)	0.91 (0.61–1.35)	53 (4.6%)	314 (3.9%)	1.19 (0.88–1.61)	1.10 (0.81–1.50)

a Adjusted for smoking status, body mass index, alchol use, history of chronic pancreatitis, history of Rheumatoid arthritis, use of other drugs, diabetes and prior cancer.

**Table 3:** Dose of non-steroidal anti-inflammatory drugs (NSAID) use and risk of pancreatic cancer

**Period of NSAID use**									
**5 years before index date, excluding the year before index date**					**Until 1 year before index date**				
**NSAID dose (DDDs)**	**Cases (*n*)**	**Controls (*n*)**	**OR (95% CI)**	**Adjusted OR (95% CI)** a	**NSAID dose (DDDs)**	**Cases (*n*)**	**Controls (*n*)**	**OR (95% CI)**	**Adjusted OR (95% CI)** a
No use: 0	534	3776	1.0	1.0	No use: 0	345	2565	1.0	1.0
Category 1: 0–9.20	156	1058	1.04 (0.86–1.26)	0.98 (0.80–1.20)	Category 1: 0–31.5	192	1354	1.06 (0.88–1.28)	1.01 (0.83–1.23)
Category 2: 9.25–26.50:	145	1059	0.97 (0.80- 1.18)	0.91 (0.74–1.11)	Category 2: 31.55–90.5	217	1357	1.20 (1.00–1.43)	1.11 (0.91–1.34)
Category 3: 26.55–100.50:	160	1020	1.11 (0.92- 1.35)	1.04 (0.85–1.27)	Category 3: 90.55–298	198	1333	1.11 (0.92–1.35)	1.03 (0.84–1.26)
Category 4: >100.50	146	1041	0.99 (0.81–1.21)	0.93 (0.77–1.15)	Category 4: >298	189	1345	1.05 (0.87–1.28)	0.96 (0.78–1.18)

Abbreviation: DDD=defined daily dose.

a Adjusted for smoking status, body mass index, alcohol use, history of chronic pancreatitis, history of rheumatoid arthritis, use of other drugs (proton pump inhibitors, H_2_ antagonists, steroids, hormone replacement therapy, disease modifying anti-rheumatic drugs), diabetes and prior cancer.

**Table 4:** Duration of non-steroidal anti-inflammatory drugs (NSAID) use and risk of pancreatic cancer

**Period of NSAID use**									
**5 years before index date, excluding the year before index date**					**Until 1 year before index date**				
**NSAID duration (days)**	**Cases (*n*)**	**Controls (*n*)**	**OR (95% CI)**	**Adjusted OR (95% CI)** a	**NSAID duration (days)**	**Cases (*n*)**	**Controls (*n*)**	**OR (95% CI)**	**Adjusted OR (95% CI)** a
No use	534	3776	1.0	1.0	No use	345	2565	1.0	1.0
Category 1: 0–33.5	155	1078	1.02 (0.84–1.23)	0.97 (0.79–1.18)	Category 1: 0–40.5	204	1377	1.10 (0.92–1.34)	1.06 (0.87–1.28)
Category 2: 33.6–156.5	152	1013	1.06 (0.88–1.29)	1.01 (0.83–1.24)	Category 2: 40.6–156.5	204	1320	1.16 (0.96–1.40)	1.09 (0.90–1.33)
Category 3: 156.6–773	171	1044	1.15 (0.96–1.39)	1.08 (0.89–1.32)	Category 3: 156.6–834	214	1346	1.18 (0.98–1.43)	1.08 (0.88–1.31)
Category 4 >773	129	1043	0.87 (0.70–1.07)	0.78 (0.62–0.97)	Category 4: >834	174	1346	0.96 (0.79–1.18)	0.86 (0.69–1.06)

a Adjusted for smoking status, body mass index, alcohol use, history of chronic pancreatitis, use of other drugs (proton pump inhibitors, H_2_ antagonists, steroids ), diabetes and prior cancer.

**Table 5:** Pancreatic cancer risk according to dose and duration of non-steroidal anti-inflammatory drugs (NSAID) use since entry into General Practice Research Database (excluding the year before the index date)

	**Dose of NSAID**			
	**Low dose (<1.0 DDD per day)**			
**Duration of NSAID use (days)**	**Cases (*n*)**	**Controls (*n*)**	**OR (95% CI)**	**Adjusted OR(95%CI)** a
Non-user and use for 0–365.3	504	3560	1.0	1.0
365.3–1095.8	87	518	1.33 (1.02–1.74)	1.17 (0.88–1.56)
1095.8–1826.3	48	355	1.02 (0.73–1.43)	0.95 (0.66–1.34)
1826.3 or more	48	421	0.79 (0.56–1.11)	0.70 (0.49–0.99)
	**High dose (⩾1.0DDD per day)**			
Non-user and use for 0–365.3	705	4976	1.0	1.0
365.3–1095.8	52	332	1.11 (0.81–1.52)	1.02 (0.73–1.42)
1095.8–1826.3	18	180	0.69 (0.42–1.14)	0.67 (0.39–1.12)
1826.3 or more	24	177	0.89 (0.56–1.40)	0.85 (0.53–1.36)

Abbreviation: DDD=defined daily dose.

a Adjusted for smoking status, body mass index, alcohol use, history of chronic pancreatitis, history of rheumatoid arthritis, use of other drugs (proton pump inhibitors, H_2_ antagonists, steroids, hormone replacement therapy and disease modifying anti-rheumatic drugs), diabetes and prior cancer.
